# An Essential Regulatory Role of Downstream of Kinase-1 in the Ovalbumin-Induced Murine Model of Asthma

**DOI:** 10.1371/journal.pone.0034554

**Published:** 2012-04-13

**Authors:** Chang-Min Lee, In Duk Jung, Kyung Tae Noh, Jun Sik Lee, Jin Wook Park, Deok Rim Heo, Jun Ho Park, Jeong Hyun Chang, Il-Whan Choi, Jong-Suk Kim, Yong Kyoo Shin, Sung-Joo Park, Myung-Kwan Han, Chun Geun Lee, Won-Kyung Cho, Yeong-Min Park

**Affiliations:** 1 Department of Microbiology and Immunology, Medical Research Institute, Pusan National University School of Medicine, Yang-san, South Korea; 2 Department of Biology, College of Natural Science, Chosun University, Gwangju, South Korea; 3 Department of Thoracic and Cardiovascular Surgery, Busan Medical Center, Busan, South Korea, Yang-san, South Korea; 4 Department of Clinical Labratory Science, College of Health & Therapy, Daegu Haany University, Gyeong San, South Korea; 5 Department of Microbiology, Inje University College of Medicine, Busan, South Korea; 6 Department of Biochemistry, Chonbuk National University Medical School, Jeonju, South Korea; 7 Department of Pharmacology, Chungang University College of Medicine, Seoul, South Korea; 8 Department of Herbology, College of Oriental Medicine, Wonkwang University, Iksan, Jeonbuk, South Korea; 9 Department of Microbiology & Immunology, Chonbuk National University Medical School, Jeonju, Republic of Korea; 10 Section of Pulmonary and Critical Care Medicine, Department of Internal Medicine, Yale University School of Medicine, New Haven, Connecticut, United States of America; International Center for Genetic Engineering and Biotechnology, India

## Abstract

The downstream of kinase (DOK)-1 is involved in the protein tyrosine kinase (PTK) pathway in mast cells, but the role of DOK-1 in the pathogenesis of asthma has not been defined. In this study, we have demonstrated a novel regulatory role of DOK-1 in airway inflammation and physiologic responses in a murine model of asthma using lentiviral vector containing DOK-1 cDNA or DOK-1-specific ShRNA. The OVA-induced inflammatory cells, airway hyperresponsiveness, Th2 cytokine expression, and mucus response were significantly reduced in DOK-1 overexpressing mice compared to OVA-challenged control mice. The transgenic introduction of DOK-1 significantly stimulated the activation and expression of STAT-4 and T-bet, while impressively inhibiting the activation and expression of STAT-6 and GATA-3 in airway epithelial cells. On the other hand, DOK-1 knockdown mice enhanced STAT-6 expression and its nuclear translocation compared to OVA-challenged control mice. When viewed in combination, our studies demonstrate DOK-1 regulates allergen-induced Th2 immune responses by selective stimulation and inhibition of STAT-4 and STAT-6 signaling pathways, respectively. These studies provide a novel insight on the regulatory role of DOK-1 in allergen-induced Th2 inflammation and airway responses, which has therapeutic potential for asthma and other allergic diseases.

## Introduction

Asthma is Th2-mediated inflammatory disease characterized by airway hyperresponsiveness (AHR), and airway remodeling that results in bronchial eosinophil accumulation [1], [2]. Allergens trigger antigen-presenting cells to interact with naïve T cells [3], [4]. These events activate Th2 cells, resulting in the over-production of various Th2 cytokines, such as interleukin (IL)-4, IL-5 and IL-13, which are known to have a critical role in the differentiation in Th2 development [5], [6], [7]. Both clinical and experimental allergic inflammations lead particularly to altered blood and lung profiles of Th1 and Th2 cytokines. It has been shown that CD4^+^ Th2 cells play a pivotal role in the pathogenesis of asthma and other allergic inflammatory diseases [5], [8], [9].

The balance between Th1 and Th2 cells is tuned by the cross talk of transcription factors. One of the major transcription factors regulating the expression of Th2 cytokine is STAT6 [10], [11], [12]. The STATs have been shown to be important in the regulation of cytokines and growth factor-inducible transcription factors in immune response [13], [14]. Recent studies using STAT6-deficient mice demonstrated that phosphorylation of STAT6 and its nuclear translocation are critical for the development of Th2 cell differentiation and airway responses [10], [11]. A significant role of STAT6 in airway inflammation was further supported by findings in asthmatic patients who showed increased levels of STAT6 expression in the lung [14]. IL-4, a prototype Th2 cytokine, enhances Th2 cell development through STAT6, which activates GATA-3 genes [15], [16]. GATA3, as a downstream transcriptional factor of STAT6, plays a key role in Th2 cell development by promoting Th2 cytokine expression through binding to a variety of regulatory regions of Th2 cytokines [16], [17]. On the other hand, IL-12 drives Th1 cell differentiation through activation of STAT 4 and T-box expressed in T cells (T-bet), Th1 transcription factor, which up-regulates IFN-γ and down-regulates IL-4 and IL-5 production [12], [17], [18].

Downstream of tyrosine Kinase-1 (DOK-1) is a common substrate of many protein tyrosine kinases (PTKs) [19], [20], [21], [22]. This is a recently discovered family of adapter molecules which have emerged as an expanding group of insulin receptors substrates-related signaling molecules, consisting of NH_2_-terminal tandem of PH and PTB domains [20], [23], [24]. DOK-1, initially designated as DOK or p62^dok^, was first branded as a major substrate of p210^bcr-abl^. Which tyrosine phosphorylation, DOK-1 and its closet homologue DOK-2 act as adaptor proteins and recruit multiple SH2-containing molecules such as rasGAP and Nck [25]. Experiments with mice lacking DOK-1 or DOK-2 established an indispensable role in the negative regulation of Erk downstream of PTKs in various hematopoietic cells [21], [26]. Furthermore, the negative regulatory role of DOK-1 was validated by the expression of small interfering RNA directed against DOK-1, which enhanced activation of MAP kinase and subsequent release of arachidonic acid and TNF-α [19], [20], [23].

Although it has been shown that DOK-1 suppresses activation of antigen/FceRI-mediated signaling pathway in RBL2H3 mast cells [22], [27], the role of DOK-1 in the development of allergic inflammation and airway response has not been explored. In this study, we hypothesized that DOK-1 plays an important regulatory role in allergen-induced lung inflammation and physiologic responses. To address this hypothesis, we generated and characterized mice in which lung DOK-1 is knock-downed (KD) or over-expressed (OE) using a lentiviral transgenic system. These studies demonstrate that DOK-1 significantly inhibits allergen-induced pulmonary inflammation and airway responses by selective inhibition and stimulation of STAT6 and STAT4 signaling pathways, respectively.

## Results

### Generation of DOK-1 KD and DOK-1 OE mice

**Figure 1:**
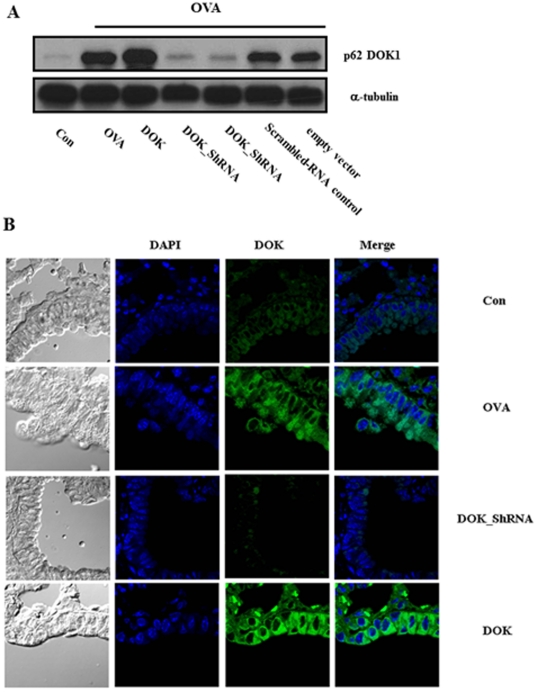
DOK-1 expression in the lungs after OVA sensitization and challenge. The lentiviral vectors containing DOK-1 specific ShRNA (DOK_ShRNA) or DOK-1 cDNA (DOK) were administrated together with controls (empty vector or vector containing non-specific scrambled ShRNA) before OVA challenge. (A) Representative western blotting demonstrating DOK-1 protein expression in the lungs of the mice. Con, non OVA challenged; OVA, OVA-challenged with empty lentiviral vector; DOK_ShRNA, OVA-challenged with DOK_ShRNA knockdown; DOK, OVA-challenged with DOK-1 overexpression (B) Immunofluorescent staining on the tissue sections from the lungs using Alexa Fluor 488-conjugated DOK-1 antibody and DAPI stains.

To understand the role of DOK-1, we generated lentiviral vector containing DOK-1- specific ShRNA and cDNA and evaluated the *in vivo* efficacy after treatment on the mice. Knockdown efficiency of 4 different DOK ShRNA in the lung was individually evaluated, and Clone 3 was selected because it showed the highest efficiency in silencing DOK-1 in the lung (>95% knockdown) (Figure 1A). Conversely, DOK is highly expressed in the lungs of mice treated with lentivirus containing DOK-1 cDNA (Figure 1A). The local tissue expression of DOK-1 was determined by confocal evaluations after DOK-1 immunostaining. We detected minimum cytosolic expression of DOK-1 in the lung epithelial cells of control mice without OVA allergen challenge (Figure 1B). However, the expression of DOK-1 was increased in the animals with OVA stimulation, and immunoreaction was remarkably negative for DOK-1 knockdown mice. On the other hand, DOK-1 overexpression was clearly appreciated in the mice treated with lentiviral transgenic construct (Figure 1B). These data demonstrate that DOK-1 expression is localized in the cytosol of airway epithelial cells, and its expression in the lung is efficiently modulated by lentiviral knockdown or transgenic approaches.

### Role of DOK-1 in OVA-induced allergic inflammation, airway responsiveness, and OVA-specific IgE production

**Figure 2 pone-0034554-g002:**
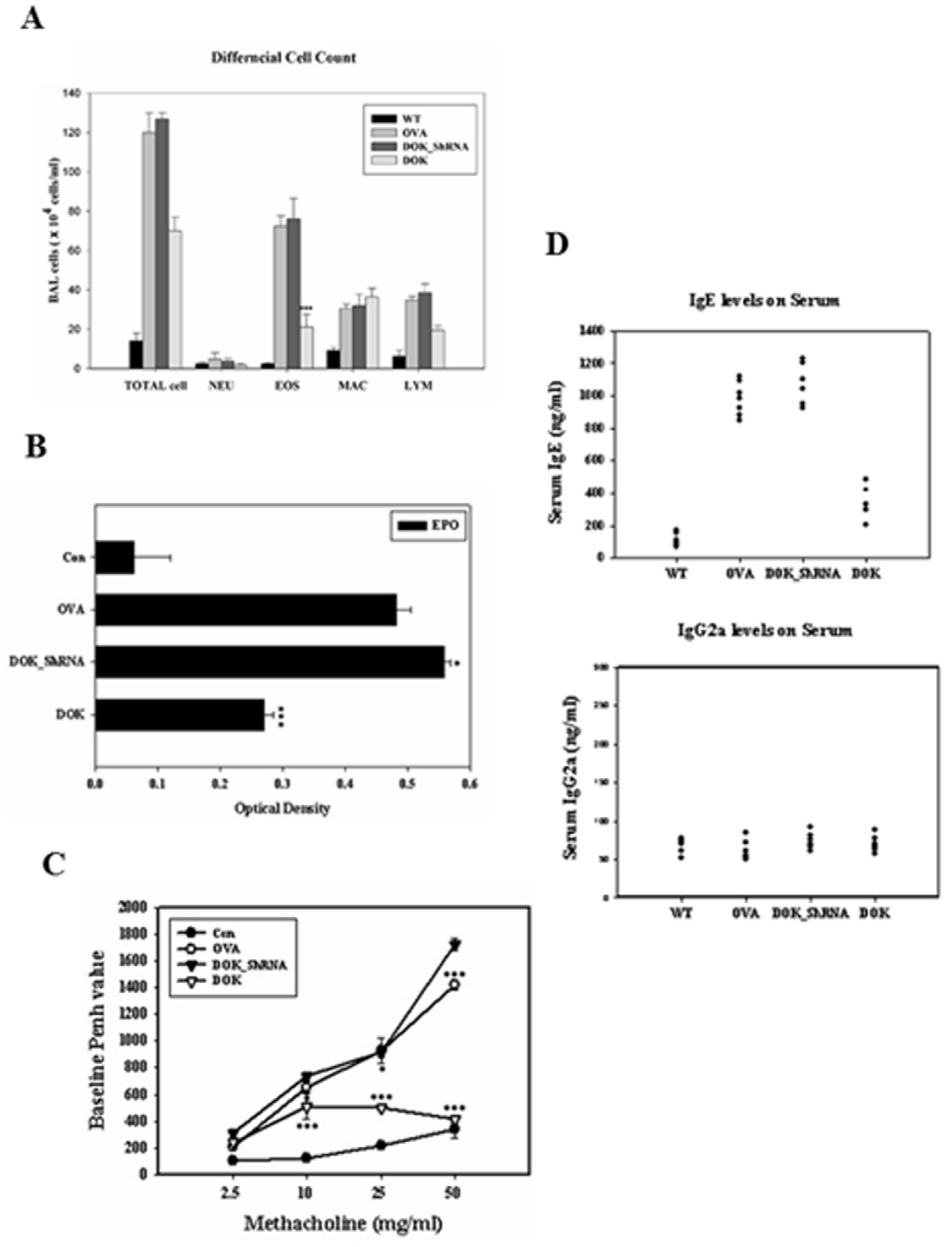
Effects of DOK-1 in OVA-induced inflammation and airway response. (A) The recovery of BAL cells 24 hr after OVA challenge. NEU, Neutrophil; EOS, Eosinophil; LYM, Lymphocyte; MAC, Macrophages; TOT, total cell. (B) Eosinophil peroxidase (EPO) activity in BAL fluids of OVA-sensitized and –challenged mice. (C) Airway responsiveness to aerosolized methacholine measured by non-invasive whole body plethysmography. (D) Serum IgE and IgG2a levels detected by ELISA. The values in all the panels represent means ± S.E.M. At least 5 mice were included in each group. *P<0.05, ***P<0.001 vs. OVA-challenged mice.

To define the role of DOK-1 in allergic inflammation, wild-type, DOK-1 knockdown (DOK_ShRNA) and transgenic mice (DOK) were sensitized and challenged with OVA. OVA sensitization and challenge significantly increased BAL inflammatory cells compared to sham controls (Figure 2A). The OVA-induced BAL inflammation was not significantly modulated in the DOK_ShRNA mice (Figure 2A), but there were slightly increased eosinophil peroxidase (EPO) levels compared to OVA-challenged mice. In contrast, DOK-1 OE mice had significantly less BAL inflammatory responses, especially in eosinophils and lymphocytes numbers, together with EPO levels. (Figure 2A and B). To determine the role of DOK-1 in allergen-induced physiologic response, we evaluated airway hyperresponsiveness (AHR) after methacholine challenge. As we expected, OVA challenge significantly increased AHR in a dose response manner and the DOK_ShRNA mice showed comparable levels of airway responsiveness. In contrast, OVA-induced AHR was abolished in the DOK-1 transgenic mice (Figure 2C). Systemic sensitization with OVA increased the serum levels of OVA-specific IgE and IgG2a. DOK-ShRNA mice showed comparable levels of serum IgE (1007.3±211.3 ng/ml) compared with OVA-treated control mice (913.2±176.51 ng/ml). On the other hand, the OVA-induced IgE levels were dramatically decreased in DOK-1 transgenic mice (323.33±94.25 ng/ml). Interestingly, IgG2a levels were not significantly changed in any of these groups of mice.

### Role of DOK-1 in OVA-induced tissue responses

**Figure 3 pone-0034554-g003:**
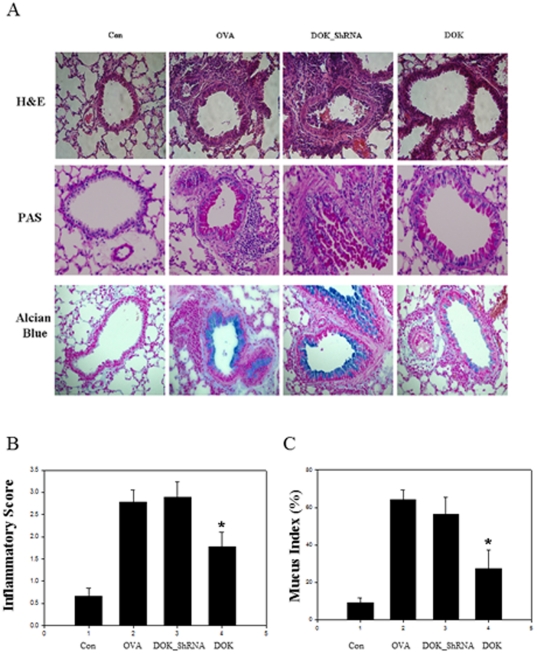
Effect of DOK-1 in OVA-induced tissue inflammation and mucus responses. (A) Lung sections were stained with hematoxylin and eosin, D-PAS, alcian blue for the evaluation of inflammatory cells and airway mucus responses. ×40 of original magnification. Con, non OVA challenged; OVA, OVA-challenged with empty lentiviral vector; DOK_ShRNA, OVA-challenged with DOK_ShRNA knockdown; DOK, OVA-challenged with DOK-1 overexpression. At least 4 mice were included in each group. (B) Inflammatory index that scored parenchymal inflammation. At least 4 mice were included in each group. *P<0.05 (C) Mucus index evaluated by morphometric analysis representing alcian blue stained mucus cells (percentage of positive cells) in airway epithelial cells. At least 4 mice were included in each group. *P<0.05.

The effects of DOK-1 in tissue inflammatory and mucus responses were evaluated by histologic examination of lung sections. Impressive perivascular and peribronchial inflammation and mucus metaplasia was noted in the mice with OVA challenge. DOK-1 knockdown mice showed comparable levels of inflammation and mucus responses compared with OVA stimulated control mice (Figure 3A). In contrast, OVA-induced inflammatory and mucus responses were significantly reduced in the DOK-1 transgenic mice (Figure 3A). In accordance with these histologic changes, the inflammatory score (Figure 3B) and mucus index (Figure 3C) showed overexpression of DOK-1 significanlty reduced OVA-induced inflammation and mucus production (*p<0.05).

### Role of DOK-1 in T cells and cytokine expression

**Figure 4 pone-0034554-g004:**
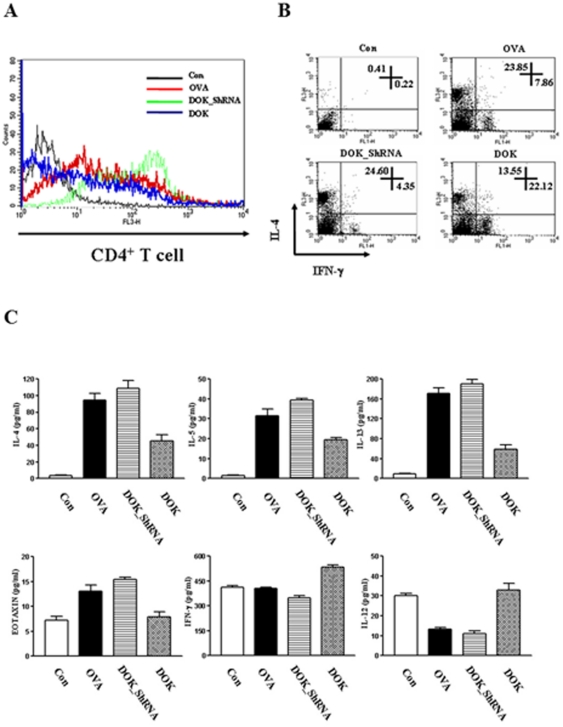
Effect of DOK-1 on T cells and cytokine/chemokine expression. (A) FACS Histogram analysis on BAL cells from the mice after OVA sensitization and challenge. Con, non OVA challenged; OVA, OVA-challenged with empty lentiviral vector; DOK_ShRNA, OVA-challenged with DOK_ShRNA knockdown; DOK, OVA-challenged with DOK-1 overexpression. Each lane indicates CD4^+^ T cell population stained with Cy5-anti-CD4 antibody. (B) BAL CD4(+)T cells gated with PE-conjugated CD4 were further evaluated by intracellular staining against Cy5-conjugated-IL-4 and FITC-conjugated-IFN-γ. (C) The levels of inflammatory Th1 and Th2 cytokines and a chemokine in BAL fluid were measured by ELISA at 24 hrs after the last OVA challenge. Data represent means ± S.E.M. Each group contains at least 7 mice. *P<0.05, ***P<0.001 vs. OVA-challenged mice.

To understand the mechanism of DOK-1 regulation in OVA-induced inflammation, we characterized T cell population and measured levels of Th1 and Th2 cytokines in the BAL fluid. Proliferative responses revealed pulmonary CD4^+^ T cell population was significantly up-regulated in DOK-1 knockdown mice compared to OVA-challenged control mice (Figure 4A). Intracellular cytokine staining of IFN-γ and IL-4 on CD4^+^ T cells isolated from BAL cells further revealed that transgenic expression of DOK-1 significantly reduced IL-4^+^ CD4^+^ T cells with increased number of IFN-γ producing CD4^+^ T cells (Figure 4B). Although the total number of cells was not significantly increased in the OVA-challenged mice with shRNA silencing of DOK-1 (Table 1), there was a significant increase in the CD4^+^ cells compared to OVA-challenged and vehicle-treated mice (Figure 4A). Interestingly, these cells are not typical Th1 or Th2 cells because the expression of IL-4 and IFN-γ was not significantly changed in the lungs from this group of mice compared to OVA challenged and vehicle treated mice (Figure 4B). Further characterization of these cells remains to be determined in future studies. On the other hand, the expression levels of IL-4, IL-5, IL-13 and eotaxin were also significantly down-regulated in DOK-1 transgenic mice compare to OVA-challenged control mice or DOK-1 KD mice. In contrast, the levels of IFN-γ and IL-12 in this group of mice were higher than OVA-challenged control mice or the DOK-1 KD mice (Figure 4C). These studies demonstrate that overexpression of DOK-1 not only suppresses allergen-induced Th2 inflammation but also stimulates IFN-γ producing Th1 cells.

**Table 1:** Inflammatory cell counts in the lungs from mice challenged with OVA and DOK-1 shRNA.

	Total cell	CD4(+) cell	IL-4(+) cell	IFN-γ (+) cell
Con	217860±10786	4571±112	326±70	237±61
OVA	1210638±16088	210651±4750	6377±419	2617±213
DOK_ShRNA	1384283±18549	307310±3515	6360±355	2339±497
DOK OE	790099±9963	119304±6197	4466±340	5295±535

### Role of DOK-1 on STAT-6 and STAT-4 signaling

**Figure 5 pone-0034554-g005:**
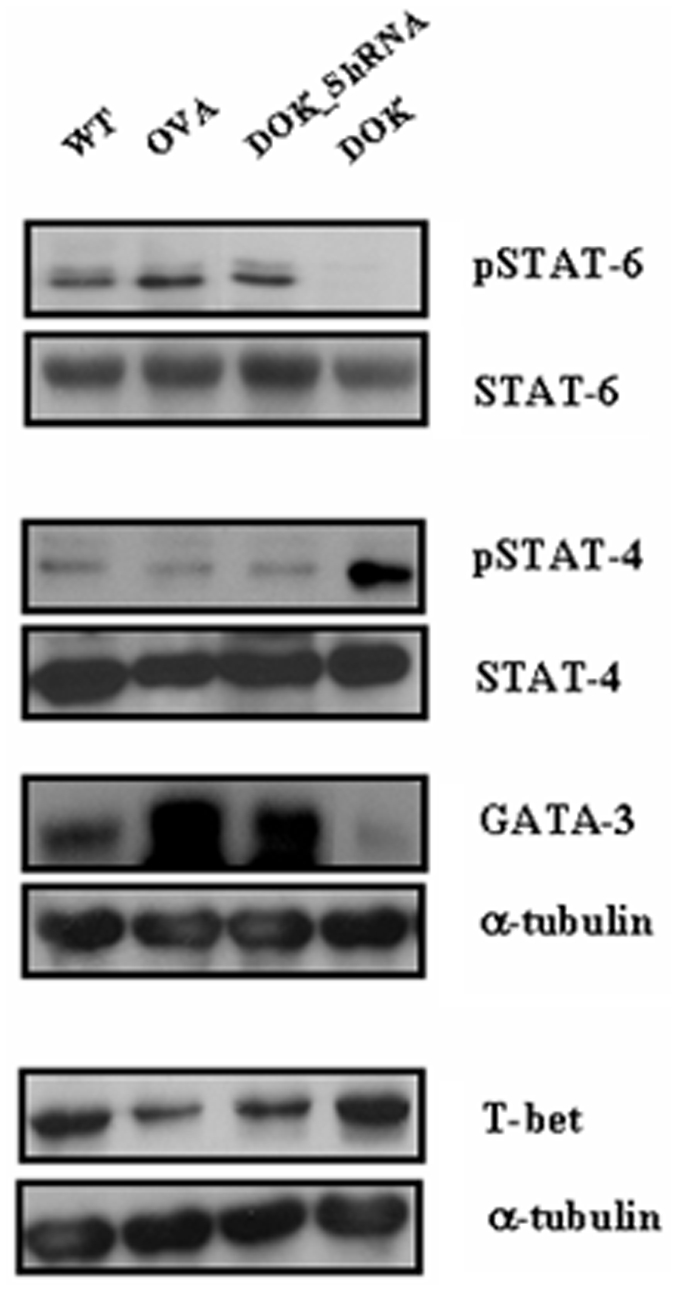
Effect of DOK-1 on STAT-6 and STAT-4 signaling pathways. Expression and/or activation (phosphorylation) of STAT6, STAT4, GATA3, and T-bet transcriptional factors were evaluated by Western blot analysis. A representative gel photo out of 6 similar independent experiments. Con, non OVA challenged; OVA, OVA-challenged with empty lentiviral vector; DOK_ShRNA, OVA-challenged with DOK_ShRNA knockdown; DOK, OVA-challenged with DOK-1 overexpression.

**Figure 6 pone-0034554-g006:**
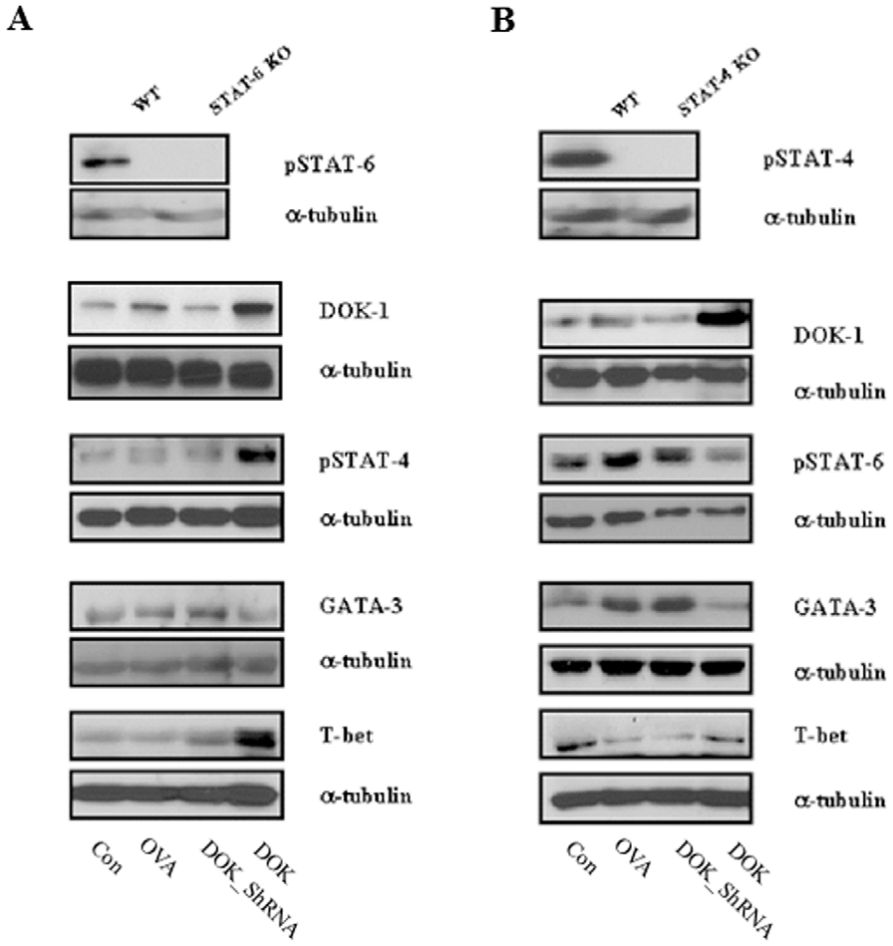
DOK-1 regulation of STAT-4, STAT-6 signaling pathways (A) Expression and/or activation of DOK-1, STAT-4, GATA-3 and T-bet in STAT-6 knockout and (B) DOK-1, STAT-6, GATA-3 and T-bet in STAT-4 knockout mice were evaluated by Western blot analysis. A representative gel photo out of five similar independent experiments. Con, non OVA challenged; OVA, OVA-challenged with empty lentiviral vector; DOK_ShRNA, OVA-challenged with DOK_ShRNA knockdown; DOK, OVA-challenged with DOK-1 overexpression.

To define the role of DOK-1 in the Th1/Th2 immune signal pathway, we evaluated the activation or expression of STAT-6, STAT-4, GATA-3 and T-bet in each group of mice. Western blot analysis revealed that DOK-1 transgenic expression significantly reduced STAT-6 activation and GATA-3 protein levels, while stimulating STAT-4 activation and T-bet expression (Figure 5). We further revealed that the regulatory affect of DOK-1 on STAT-6 and STAT-4 signaling pathways are mostly independent from each other, because similar regulation was observed both in STAT6 and STAT4 null mice. In the absence of STAT-6, DOK-1 OE significantly activates STAT-4 and T-bet expression (Figure 6A). Similarly, in the absence of STAT-4, the DOK-1 inhibition of STAT-6 activation and GATA3 expression was not significantly affected (Figure 6B). Thus, these studies indicate that DOK-1 has the capacity to selectively activate and inhibit STAT6 and STAT4 signaling pathways, respectively, in the regulation of allergen-induced Th2 immune responses.

### Role of DOK-1 in STAT-6 and STAT-4 expression and nuclear translocation

**Figure 7:**
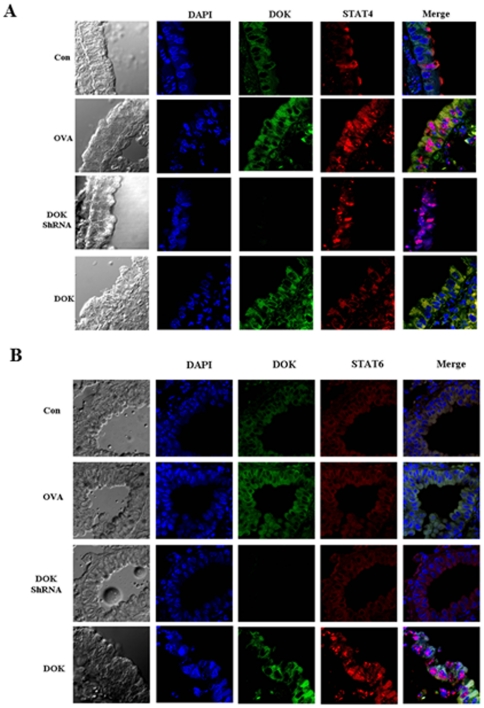
Effects of DOK-1 on STAT-6 and STAT-4 expression and nuclear translocation in airway epithelial cells. (A) Lung sections were stained with Alexa Fluor 488-conjugated DOK-1 and Alexa Fluor 568-conjugated STAT-6 antibodies and DAPI stain. (B) Lung sections were stained with Alexa Fluor 488-conjugated DOK-1 and Alexa Fluor 568-conjugated STAT-4 antibodies and DAPI stains. A representative photo of 7 similar experiments. Con, non OVA challenged; OVA, OVA-challenged with empty lentiviral vector; DOK_ShRNA, OVA-challenged with DOK_ShRNA knockdown; DOK, OVA-challenged with DOK-1 overexpression.

In non-stimulated sham control mice, we detected only minimal levels of cytosolic expression of STAT-4 and STAT-6 in airway epithelial cells by immunohistochemistry. However, after OVA challenge, we noted significant nuclear translocation of STAT-4 and STAT-6 from cytoplasm. The STAT-6 expression and nuclear translocation were further increased in the OVA-challenged DOK-1 KD mice (Figure 7A). However, DOK-1 OE mice showed significantly reduced the expression and translocation of STAT-6 into nucleus (Figure 7A). On the other hand, DOK-1 OE mice showed significantly increased STAT-4 expression and translocation into nucleus (Figure 7B).

## Discussion

Murine models of asthma have been used to understand the pathogenesis of human asthma [28]. In this study, we demonstrate a novel regulatory role of DOK-1 in OVA-induced allergic inflammation and airway responses by letiviral-mediated DOK-1 knockdown and DOK-1 transgenic mice. To our knowledge, this study is the first to provide experimental evidence demonstrating the regulatory role of DOK-1 in allergen-induced airway inflammation and physiologic responses in a murine model of asthma.

Asthma is an inflammatory disease characterized by Th1/Th2 balance that can proceed to life-threatening airway obstruction [29], [30], [31]. Th2 cytokines, IL-4, IL-5 and IL-13 produced by activated CD4^+^ T cells, play a central role in the pathogenesis of asthma by controlling the key process of immunoglobulin E (IgE) production, growth of mast cells and the differentiation and activation of mast cells and eosinophils [32], [33], [34]. In contrast, Th1 cytokines such as IFN-γ and IL-12, which down-regulate Th2 responses inhibit the development of allergic lung inflammation [35], [36], [37]. Thus, interventions that inhibit Th2 cytokines by enhancing Th1 cytokine production may be useful for the treatment of allergic asthma.

Th2 cytokines play a pivotal role in the induction, and regulation of allergic diseases by regulating IgE production, differentiation of naïve T cells toward Th2 cells and activation of mast cells. The critical role of STAT-6 and GATA-3 in IL-4 signaling has been demonstrated in STAT-6 deficient mice. In the absence of STAT-6, mice did not develop Th2 responses, Ig class switching to IgE, AHR, mucus hypersecretion and airway eosinophilia upon allergen sensitization and airway challenges, highlighting the essential role of this transcription factor in the pathogenesis of allergic asthma [16], [38].

DOK-1 negatively regulates Ras-Erk signaling and functions as an intermediary adaptor protein in this pathway [39]. Recent studies further demonstrated that DOK-1 associates with CD45 in a tyrosine phosphorylation dependent manner and negatively regulate JAK/STAT pathways [25]. Moreover, site-directed mutagenesis led to the identification of tyrosine residue (Y296) in DOK-1 as a pivotal site for CD45/DOK-1 interaction. Upon anti-CD3/TCR stimulation, DOK-1 translocates from the cytoplasm to the plasma membrane to serves as a downstream effector of CD45, and negatively regulates JAK/STAT pathways [25]. Together with DOK-2, DOK-1 also negatively regulates lipopolysaccarid stimulated TLR-4 signaling pathways [26]. In addition, recent studies further demonstrated that DOK-1 regulates specific immune cell function such as mast cells [22]. However, the in vivo role of DOK-1 in allergic inflammation and airway response has not been defined.

As an animal model of asthma, the mice sensitized and challenged with OVA developed Th2 inflammation, mucus metaplasia and airway hyperresponsiveness, hallmarks of asthmatic airways. Because we administrated the lentiviral vectors containing ShRNA or DOK-1 cDNA before OVA challenge, we speculated that DOK-1 modulates effector function of immune cells or other mediators. In particular, we noted significant changes in the STAT-6 and STAT-4 signaling molecules in airway epithelial cells with genetic modification of DOK-1. In accord with this finding, a number of previous studies demonstrated that airway epithelial cells play a critical role in allergen-induced inflammation and physiologic response [40], [41]. Present studies clearly indicate that transgenic introduction of DOK-1 significantly down-regulates allergen-induced STAT6 expression and its activation (phosphorylation and nuclear translocation) in airway epithelial cells. This data suggest that DOK-1 plays an important role in these signaling pathways in epithelial cells, as was the case in the hematopoietic inflammatory cells. However, we could not rule out the possibility that DOK-1 primarily affects the inflammatory cells such as mast cells or dendritic cells that have the capacity to drive Th2 inflammation and tissue responses. In this regard, the specific regulatory function DOK-1 on each immune cell needs to be further defined in future studies. The effect of local administration of lentiviral vectors containing these DOK-1 transgenes also remains to be determined.

Interestingly, endogenous DOK-1 expression was increased in the mice with OVA challenge. Although it did not reach statistical significance, knock down DOK-1 showed a trend of increase in BAL inflammation and airway responsiveness, especially in EPO (+) cells and airway responsiveness at 50 mg/ml methacholine challenge, compared to vehicle-challenged mice (Figure 2). Thus, it is reasonable to speculate that the increased expression of endogenous DOK-1 after OVA challenge is a partially protective response that limit exaggerated responses to OVA allergen challenge. Because we only noted significant suppression of allergic responses with overexpression of DOK-1, we speculate that the level of OVA-induced endogenous DOK-1 expression was not enough to suppress the allergic response itself. We did not see any significant inflammatory and histologic changes in the naive mice with DOK-1 silencing.

In this study, transgenic introduction of DOK-1 impressively modulated OVA-induced CD4 T cell recruitment to BAL and lungs and serum IgE levels. Interestingly, the total number of cells in the lung was not significantly increased in the OVA-challenged mice with shRNA silencing of DOK-1, but this group of mice showed a significant increase in CD4(+) cells compared to OVA-challenged and vehicle-treated mice. Further FACS evaluation indicated possible involvement of non-Th1 or Th2 cells in these mice, because no significant changes in the expression of Th2 (IL-4 and IL-13) or Th1 (IFN-γ) cytokines in these group mice were noted compared to OVA-challenged and vehicle treated mice. The potential contribution of these cells into allergic response remains to be determined in future studies. On the other hand, the mice treated with lentiviral vectors with DOK-1 transgene significantly increase in the number of OVA-induced Th1 cells (IFN-γ(+), IL-4(−)) and a reduction in the number of Th2 cells (IL-4(+), IFN-γ (−)). These studies suggest that DOK-1 strongly stimulates Th1 while suppressing Th2 polarization after antigen stimulation. The enhanced activation and expression of STAT-4 and T-bet, the exclusive transcriptional factors for Th1 differentiation, by DOK-1 transgene mechanistically support T cell differentiation by DOK-1. Although there could be a reciprocal interaction between STATs over transcriptional cofactors such as CBP/p300, DOK-1 regulation of STAT-4 and STAT-6 are mostly independent of each other, because STAT-6 or GATA-3 suppression by DOK-1 were not significantly modulated in the absence of STAT-4. The mechanism underlying selective activation and inhibition of STAT-6 and STAT-4 will be important in understanding the role of DOK-1, especially in immune cell functions. Further cell –specific regulatory mechanisms of DOK-1 on STAT signaling remains to be determined in future studies.

In our current studies, we did not note compromised IgE response in the mice treated with shRNA silencing of DOK-1, in contrast to the results shown in Dok-1 null mice by Yamanashi et al [21]. The expression of endogenous DOK-1 was partially suppressed in the cells with siRNA silencing. On the other hand, in DOK-1 null mutant mice, all the cells including immune cells lack DOK-1 expression from the developmental stage. We speculate that the extent of DOK-1 knock down in a variety of immune cells in vivo could be a major reason for the difference in the immune cell responses between these two studies. In addition, we could not completely exclude the possibility of inherent functional deviation of immune cells in the DOK-1 null mice affecting the overall allergen sensitization, processing and effector function of a variety of immune cells. Currently, it is not clear whether the impaired IgE response seen in DOK-1 null mice after allergen challenge resulted from the impaired function of B cells or other immune cells related to antigen processing. To address these controversial issues in DOK-1 null mutant mice, the use of conditional DOK-1 null mice or cells will be important in future studies.

In summary, these studies demonstrate that DOK-1 is a negative regulator of allergen-induced Th2 inflammation, mucus production and airway hyperresponsiveness in a murine model of asthma. DOK-1 may regulate Th1/Th2 balance through selective inhibition and activation of STAT-6 and STAT-4 signaling pathways. These studies also highlight a therapeutic potential of DOK-1 in the intervention of asthma and other allergic diseases.

## Materials and Methods

### Mice and experimental protocol

Female C57/BL6 mice, 6–8 weeks of age and free of murine-specific pathogens, were obtained from the Orient (Seoul, Korea), and all experimental animals used in this study were under a protocol approved by the Institutional Animal Care and Use Committee of the Pusan National University (PNU-2009-0036). Mice were sensitized on days 1 and 15 by intraperitoneal injection of 20 µg ovalbumin (OVA) (Sigma-Aldrich, St. Louis, MO) added in 1 mg of aluminum hydroxide (Pierce Chemical Co., Rockford, IL) in a total volume of 200 µl. One week later, mice were exposed to aerosolized OVA once a day for 3 days. [42] Lentiviral vectors containing DOK-1 specific ShRNA or DOK-1 cDNA were intraperitoneally injected on days 18 and 19. The mice were sacrificed and evaluated 24 hrs after the last OVA challenge. (Figure S1)

### Construction of lentiviral vector containing DOK-1 ShRNA and cDNA

Four ShRNAs target sequences (Clone 1: TCRN0000088814, clone 2: TCRN0000088815, clone 3: TCRN0000088816, clone4: TCRN0000088817) were selected for DOK-1 knockdown experiments (Mission ShRNA, Sigma-Aldrich, St. Louis, MO). DOK-1 cDNA was cloned by PCR amplification using the following primers : 5′-CTACCTGAGCTACCAGTCCGC-3′ (Sense) and 5′-CGTGAAGAATGTGCGAGAC-3′ (antisense) (Genecopoeia, Germantown, MD). DOK-1 ShRNAs and cDNA were further cloned into pLKO.1-puro and transferred to 293FT cells using calcium phosphate-mediated transfection. Then the viruses were collected and concentrated 100-fold by ultra-centrifugation. The virus stocks were titrated using 293FT cell-based GFP expression assay and a viral titer of 1×10^6^ IFU was used in this study.

### Evaluation of BAL cells

The total cell numbers were counted with a hemocytometer. Smears of BAL cells prepared with Cytospin II (Shandon, Runcorn, UK) were stained with Diff-Quik solution (Dade Diagnostics of P.R. Inc, Aguada, PR) for differential cell counting. Two independent, blinded investigators counted the cells, using a microscope. Approximately 200 cells were counted in each of four different random locations.

### Th1 and Th2 cell evaluation

BAL cells were first blocked with 10% (v/v) normal goat serum for 15 min at 4°C and stained with Cy5PE-conjugated mouse mAbs against CD4, then cells were analysed on a FACSCalibur cytometer (Becton Dickinson, Franklin Lakes, NJ). For intracellular cytokine staining, the cells were treated with brefeldin A (10 lg/ml; 4 hr), washed with 1% v/v FBS-PBS (staining buffer), stained with PE-conjugated mouse mAbs against CD4, IFN-g with FITC-conjugated and IL-4 with Cy5PE-conjugated and fixed in 4% w/v paraformaldehyde (20 min at room temperature).

### Measurement of eosinophil peroxidase

The suspension of BAL cells and the pulmonary homogenates were frozen/thawed three times using liquid nitrogen and a water bath at 37°C to obtain the EPO. The BAL fluid was centrifuged to 4°C for 10 min and serially diluted in a 96-well plate (75 ml/well) followed by the addition of 150 ml of substrate (1.5 mM o-phenylenediamine and 6.6 mM H_2_O_2_ in 0.05 M Tris-HCl, pH 8.0). After 30 min at room temperature, the reaction was stopped by the addition of 75 ml of 30% H_2_SO_4_, and the absorbance of the samples was determined at 492 nm on an ELISA reader.

### Histopathology

At 24 hrs after the last challenge, lungs were removed from the mice after they had been sacrified. Prior to the removal of the lungs, the lungs and trachea were filled intratracheally with a fixative (4% paraformaldehyde) using a ligature around the trachea. Lung tissues were fixed with 10% (v/v) paraformaldehyde. The specimens were dehydrated and embedded in paraffin. For histological examination, 4 µm sections of fixed embedded tissues were cut on a Leica model 2165 rotary microtome (Leica, Nussloch, Germany), placed on glass slides, deparaffinized, and sequentially stained with hematoxylin 2 and eosin-Y (Richard-Allan Scientific, Kalamazoo, MI), D-PAS and Alcian Blue staing kit (Merck, Washington, NJ). The tissue inflammation was scored in a blinded manner as shown previously [43]. The lung sections were taken from the same lobe in each mouse and at least 3 random sections per mouse were analyzed. A inflammation score of 0 to 4 was assigned to each section. (0: no inflammation; 1: mild; 2: moderate; 3: severe but not in all airways; 4: severe in all airways). Airway mucus levels were quantitated by mucus index by counting mucus containing airway cells as a percentage of total cells [44].

### Measurement of Th1/Th2 cytokines and OVA-specific IgE, IgG2a levels

Levels of IL-4 and IL-5 were quantified in the supernatants of BAL fluids by enzyme immunoassays performed according to the protocol of the manufacturer (IL-4, IL-5, IL-12, IFN-γ; Eotaxin R&D Systems, Inc., Minneapolis, MN). The levels of OVA-specific IgE and IgG2a in serum were assessed by enzyme immunoassays according to the manufacturer's protocol (R&D Systems; Minneapolis, MN).

### Whole body plethysmography

Airway responsiveness was measured in mice 24 hrs after the last challenge in an unrestrained conscious state, as described previously [15], . Mice were placed in a barometric plethysmographic chamber (All Medicus Co., Seoul, Korea) and baseline readings were taken and averaged for 3 min. Aerosolized methacholine in increasing concentrations (2.5 to 50 mg/ml) was nebulized through an inlet of the main chamber for 3 min. Readings were taken and averaged for 3 min after each nebulization. Enhanced pause (Penh), calculated as (expiratory time/relaxation time-1)×(peak expiratory flow/peak inspiratory flow), according to protocol of the manufacturers, is a dimensionless value that represents a function of the proportion of maximal expiratory to maximal inspiratory box pressure signals and a function of the timing of expiration. Penh was used as a measure of airway responsiveness to methacholine. Results were expressed as the percent increase of Penh following challenge with each concentration of methacholine, where the baseline Penh (after saline challenge) was expressed as 100%. Penh values were averaged for 3 min after each nebulization and evaluated.

### Western blot analysis

The lung tissues were homogenized, washed with PBS, and incubated in lysis buffer plus a protease inhibitor cocktail (Sigma, St Louis, Mo) to obtain extracts of lung proteins. The samples were loaded to 10% SDS-PAGE gels and were separated at 120 V for 90 minutes. The blots were incubated with an anti-STAT-6, STAT-4, GATA-3, T-bet antibody diluted at a ratio of 1∶1000, (Santa Cruz Biotechnology, Santa Cruz, CA) overnight at 4°C. The membranes were stripped and reblotted with a-tubulin antibody (Sigma) to verify the equal loading of protein in each lane.

### Detection of STAT-6 and STAT-4 by confocal microscopy

To prepare specimens for confocal microscopy, lungs were sectioned on 4-chamber Lab-Tek II slides (Thermo Fisher Scientific). One day later, slides were washed twice with PBS then blocked with 1% BSA in PBS for 20 minutes at room temperature. Slides were stained with 0.5 µg Alexa Fluor 488-conjugated DOK-1 and Alexa Fluor 568–labeled STAT-6 or STAT-4 in 200 µl volume for 30 minutes, washed 5 times, then fixed in 4% formaldehyde. Specimens were washed, excess moisture was removed, and slides were cover-slipped using Prolong Gold with DAPI (Invitrogen). After curing overnight, slides were sealed with acrylic nail polish. Slides were imaged using an Olympus Fluoview FV300 laser scanning confocal microscope. Exposure times for individual filter sets and gain multiplication settings were established for each experiment using unstained specimens to determine maximal settings.

### Statisitcal Evaluation

Experiments were repeated at least three times with consistent results. Unless otherwise stated, data are expressed as the mean ± S.E.M. ANOVA was used to compare experimental groups to control values, while comparisons between multiple groups were performed using Tukey's Multiple Comparison test. Statistical significance was indicated by a *P* value less than 0.05.P^***^<0.001, P^**^<0.01, P^*^<0.05.

## Supporting Information

Figure S1 **Schematic diagram of the experimental protocol.** Mice were sensitized on days 1 and 15 by intraperitoneal injection OVA added in 1 mg of aluminum hydroxide. After 1 week, mice were exposed to aerosolized OVA once a day for 3 days. Lentiviral vectors containing DOK-1 specific ShRNA or DOK-1 cDNA were intraperitoneally injected on days 18 and 19.
(TIF)
