# Supplementary figures and images for: An Essential Regulatory Role of Downstream of Kinase-1 in the Ovalbumin-Induced Murine Model of Asthma
Source: PLoS One. 2012 Apr 13;7(4):e34554. doi: 10.1371/journal.pone.0034554 (PMC3326039; doi:10.1371/journal.pone.0034554)

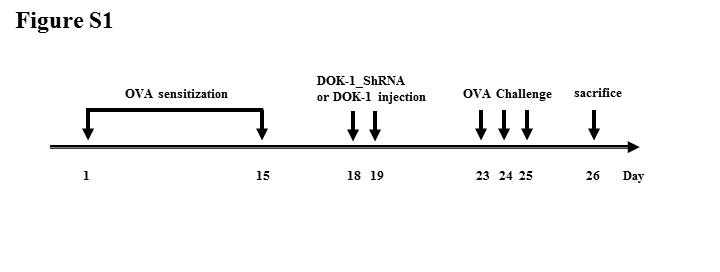

Supplement: Figure S1 — Schematic diagram of the experimental protocol. Mice were sensitized on days 1 and 15 by intraperitoneal injection OVA added in 1 mg of aluminum hydroxide. After 1 week, mice were exposed to aerosolized OVA once a day for 3 days. Lentiviral vectors containing DOK-1 specific ShRNA or DOK-1 cDNA were intraperitoneally injected on days 18 and 19. (TIF) [file pone.0034554.s001.tif]
